# Microbial Diversity in a Permanently Cold and Alkaline Environment in Greenland

**DOI:** 10.1371/journal.pone.0124863

**Published:** 2015-04-27

**Authors:** Mikkel A. Glaring, Jan K. Vester, Jeanette E. Lylloff, Waleed Abu Al-Soud, Søren J. Sørensen, Peter Stougaard

**Affiliations:** 1 Department of Plant and Environmental Sciences, University of Copenhagen, Frederiksberg, Denmark; 2 Department of Biology, University of Copenhagen, Copenhagen, Denmark; Belgian Nuclear Research Centre SCK•CEN, BELGIUM

## Abstract

The submarine ikaite columns located in the Ikka Fjord in Southern Greenland represent a unique, permanently cold (less than 6°C) and alkaline (above pH 10) environment and are home to a microbial community adapted to these extreme conditions. The bacterial and archaeal community inhabiting the ikaite columns and surrounding fjord was characterised by high-throughput pyrosequencing of 16S rRNA genes. Analysis of the ikaite community structure revealed the presence of a diverse bacterial community, both in the column interior and at the surface, and very few archaea. A clear difference in overall taxonomic composition was observed between column interior and surface. Whereas the surface, and in particular newly formed ikaite material, was primarily dominated by *Cyanobacteria* and phototrophic *Proteobacteria*, the column interior was dominated by *Proteobacteria* and putative anaerobic representatives of the *Firmicutes* and *Bacteroidetes*. The results suggest a stratification of the ikaite columns similar to that of classical soda lakes, with a light-exposed surface inhabited by primary producers and an anoxic subsurface. This was further supported by identification of major taxonomic groups with close relatives in soda lake environments, including members of the genera *Rhodobaca*, *Dethiobacter*, *Thioalkalivibrio* and *Tindallia*, as well as very abundant groups related to uncharacterised environmental sequences originally isolated from Mono Lake in California.

## Introduction

Extremophiles are organisms that grow and reproduce optimally at or near the extreme ranges of environmental variables. This can be extremes of temperature, pressure, pH, salinity, aridity or radiation and environments representing one or more of these variables are common on earth. The largest proportion of such extreme habitats is comprised of permanently cold areas, including polar regions, permafrost, deep oceans, and alpine regions. Similarly, naturally occurring stable alkaline environments such as soda lakes and deserts, and alkaline ground water are distributed globally, although these occur much less frequently. A large number of bacteria have been isolated from these environments and they often show adaptations to optimal growth under the prevailing conditions [1–3].

Soda lakes are the most thoroughly studied natural alkaline environment in terms of microbial diversity. They are mainly confined to arid regions at temperate and tropical latitudes and are characterised by high concentrations of sodium carbonate and sodium chloride, which is reflected in the adaptations of microorganisms isolated from these environments [4–6]. Primary production from cyanobacteria and anoxygenic phototrophic bacteria is very high and supports a diverse alkaliphilic microbial community with representatives of the major trophic groups of archaea and bacteria participating in cycling of carbon, nitrogen, and sulphur under aerobic and anaerobic conditions [2, 5–7]. Terrestrial serpentinising sites represent another stable alkaline environment with low-salinity. Serpentinisation is the reaction of water with ultramafic minerals to form a highly alkaline environment rich in hydrogen and methane and low in inorganic carbon, making the geochemistry distinct from the characterised soda lakes. The microbial diversity at a few such sites have been described, including the subterrestrial Cabeço de Vide Aquifer in Portugal [8, 9] and serpentinite spring water from The Cedars in northern California [10], the Tablelands Ophiolite in Newfoundland, Canada [11] and Maqarin, Jordan [12], and bacteria of the class *Clostridia* and hydrogen-utilising *Betaproteobacteria* related to the genus *Hydrogenophaga* are abundant in serpentinite water [9–11, 13].

Permanently cold, stable alkaline environments are a very rare occurrence and only a few such environments have been described: The upper layers of the permanently ice-covered Lake Untersee in east Antarctica [14], a series of alkaline ponds in the McMurdo Dry Valley Region in the Antarctica [15], and the unique submarine ikaite tufa columns located in the Ikka Fjord in Southern Greenland [16]. The ikaite columns represent a permanently cold (less than 6°C) and alkaline (pH > 10) environment with a salinity of less than 10 ‰ [16–18]. The columns are composed of a metastable hexahydrate of calcium carbonate, called ikaite, a rare low-temperature mineral named after the location where it was first described. Although ikaite can be found as microscopic and macroscopic crystals in many cold marine environments [19–21], the Ikka Fjord is the only known location where larger structures of ikaite are formed. The columns grow from the bottom of the shallow inner fjord and are formed where alkaline submarine spring water rich in sodium carbonate mixes with the cold and calcium-rich seawater of the Ikka Fjord. Ikaite precipitation is favoured by the low temperatures and high phosphate content of the spring water. Column growth is primarily vertical and they may reach heights of up to 20 m and several meters in diameter [16, 18]. Apart from sodium, phosphate, and dissolved inorganic carbon, the column seep water contains low concentrations of inorganic compounds compared to the surrounding seawater [18]. The level of dissolved organic carbon is unknown, but the rich fauna and flora covering the columns combined with their vertical growth has led to speculations that the heterogeneous appearance of some column cross sections could be caused by trapped organic matter [22–24]. According to previous reports, the interior of the ikaite columns is home to a variety of cold- and alkaline-adapted bacteria [25, 26] including the to date only characterised bacterium displaying both psychrophilic and alkaliphilic growth properties, *Rhodonellum psychrophilum* [27, 28], and the columns have been the target of recent bioprospecting studies aimed at identifying cold- and/or alkaline-adapted enzymes [29–32].

There is significant biotechnological interest in microorganisms and enzymes from cold and alkaline environments and numerous studies have focussed on the isolation and characterisation of novel enzymes for low temperature and/or high pH applications [33–36]. In addition to the biotechnological potential, studies of the microbial community inhabiting the ikaite columns may yield insights into the mechanisms and adaptations that allow life to thrive under these conditions and specifically, how they differ from the temperate and/or high-saline alkaline environments. In this study, we used pyrosequencing of 16S rRNA genes to carry out an extensive characterisation of the bacterial and archaeal communities inhabiting the cold and alkaline ikaite columns and the surrounding Ikka Fjord. The results demonstrate that the columns are inhabited by a diverse bacterial community and identify several close relatives of bacterial groups and characterised alkaliphiles known to inhabit soda lake environments. Together with the unusual environmental conditions, these findings highlight the ikaite columns as a unique resource for both evolutionary studies and future bioprospecting projects.

## Materials and Methods

### Sample collection in the Ikka Fjord

Samples of ikaite columns were collected from two locations in the Ikka Fjord, the Atoll Field and the Camp Field [18], during the summer of 2006, 2007, 2010 and 2011. Permission to sample in the Ikka Fjord was covered by a survey license granted by the Ministry of Industry and Mineral Resources, Government of Greenland. Columns were selected based on differences in size and apparent age and ikaite material was collected at depths from 5–10 m by sawing off the top 20–50 cm from individual columns. Column material was kept at 4°C for up to 48 h until subsampling could be carried out. Additional samples were taken from previously collected frozen sections kept at -20°C. Samples of ikaite material for DNA extraction were taken from the surface and cross-sections of columns by drilling 2–3 cm deep holes with a sterile 5 mm drill. For samples taken from cross-sections, the first 5 mm were discarded to minimise contamination from seawater introduced during the underwater sampling. The expected high pH in the cross-sections was confirmed by pH strips before sampling. Surface samples of newly formed, soft ikaite were taken with a spatula by scraping of the top 5 mm of a 1–2 cm diameter area. All samples were homogenised as part of the drilling procedure or by stirring with a spatula. Seawater samples were taken at 8–10 m depth and 1 l of water was filtered through a 0.22 μm filter, which was subsequently frozen at -20°C. A recent long-term underwater study measured a summer temperature of 3–5°C and a mean pH of 8.1 at this depth [17]. Sediment material was collected as a single sample of the top 10 cm layer at the Atol and Camp Field. Three separate subsamples were subsequently taken from each location before DNA extraction. All samples were stored at -20°C until DNA extraction.

### DNA extraction, PCR amplification of 16S rRNA genes and pyrosequencing

A total of 70 ikaite samples, 3 seawater samples, and 6 sediment samples were used for DNA extraction. DNA was extracted from 0.5 g ikaite or sediment material using a ceramic bead beating procedure with the MO-BIO Powersoil DNA Extraction Kit (MO-BIO Laboratories, Carlsbad, CA, USA) modified with G1 blocker (Carsten Suhr Jacobsen, GEUS, Denmark). DNA from seawater samples were extracted using the same method after homogenisation of filters in liquid nitrogen. A 466 bp fragment covering the V3 and V4 hypervariable regions of the 16S rRNA gene from bacteria and archaea was PCR amplified using the primers 341F (5’-CCTAYGGGRBGCASCAG-3’) and 806R (5’-GGACTACNNGGGTATCTAAT-3’) [37]. The amplification efficiency of this primer pair, evaluated using the TestPrime function in the SILVA ribosomal RNA database (http://www.arb-silva.de/; database version SSU r121), was 70.7% and 68.8% for a complete match and 77.6% and 76.3% allowing one mismatch (excluding the three nucleotides in the 3’ end of the primers) for domain *Bacteria* and *Archaea*, respectively. The PCR amplification and preparation of amplicon libraries were performed essentially as described [38]. Briefly, PCR reactions (50 μl) included 5 ng of template DNA, 1 U of Phusion HotStart DNA polymerase (Finnzymes, Vantaa, Finland), 1x Phusion HF Buffer, 200 μM of each dNTP and 0.5 μM of each primer and were amplified with the following cycle conditions: 98°C for 30 s, followed by 30 cycles of 98°C for 5 s, 56°C for 20 s and 72°C for 20 s and a final extension of 72°C for 5 min. PCR products were separated on a 1% agarose gel and purified using an E.Z.N.A. Gel Extraction Kit (Omega Bio-Tek, Norcross, GA, USA) and quantified using the Quant-iT dsDNA HS Assay Kit (Invitrogen, Life Technologies Europe, Naerum, Denmark). PCR products were successfully obtained for all seawater and sediment samples and for 48 of the 70 ikaite samples (S1 Table). The PCR products from the three sediment DNA extractions from each location were pooled in equal amounts to give one sample per location before further processing. Two technical controls were prepared by three separate PCR amplifications of DNA from samples I11 and I39.

Adapters and tags for pyrosequencing were added in a second 10-cycle PCR on 5 ng of purified PCR product using the conditions described above with primers 341F and 806R carrying sequencing adapters and tags for multiplexing. The amplified fragments were gel-purified, quantified, and mixed in equal amounts before sequencing on a Genome Sequencer FLX pyrosequencing system (454 Life Sciences, Roche, Branford, CT, USA).

### Sequence analysis

Trimming and quality-filtering of 16S sequences was performed using the software suite Biopieces (www.biopieces.org). Initially, tags for multiplexing and primer sequences used in the initial PCR (341F and 806R) were removed, discarding any sequences that did not contain both a multiplexing tag and the forward primer sequence. Sequences were then trimmed from both ends at the first high-quality base (Phred quality score of 25) and sequences with an average Phred quality score lower than 25 were discarded. Finally, sequences shorter than 250 bases and containing more than one ambiguous nucleotide were discarded. The complete dataset of quality-filtered sequences in QIIME-compatible format is available from the MG-RAST server (http://metagenomics.anl.gov/; ID 4587481.3).

OTU clustering was performed using USEARCH [39], which included: 1. Dereplication and subsequent error-correction by outputting the consensus sequences of an initial clustering-step at 97% identity. 2. Removing chimeric sequences using UCHIME [40] by comparison to the chimera-free Greengenes database (version 13_05; http://greengenes.lbl.gov/) [41] clustered at 97% identity. 3. A final OTU clustering step at 97% identity. The parameters for chimera detection were set to ignore low-divergence chimeras by disregarding OTUs with at least 97% identity to a sequence in the database and increase sensitivity to higher-divergence chimeras (UCHIME parameters:-minh 0.22-mindiv 3-mindiffs 5).

Phylogenetic analysis was performed using the Quantitative Insights into Microbial Ecology (QIIME) pipeline version 1.7 (www.qiime.org) [42]. The USEARCH OTUs were used as a reference set for USEARCH-based OTU picking in QIIME using the original sequences as input. Since OTUs containing only one sequence (singletons) have a much higher chance of representing PCR and sequencing errors or low-level contaminants, and are less likely to be ecologically important, these were removed before subsequent analysis to improve the overall quality of the dataset. The most abundant sequence from each OTU was chosen as a representative dataset and aligned with PyNAST in QIIME. Sequences aligning with less than 70% identity were discarded as likely non-16S contaminants and a phylogenetic tree was constructed.

Taxonomy was assigned using the RDP classifier with a confidence threshold of 50% [43] and a training set from the Greengenes database (version 13_05). Rarefaction curves, the Shannon diversity index, and clustering analysis were performed using the QIIME scripts alpha_diversity.py and beta_diversity_through_plots.py with default options.

## Results and Discussion

### Sampling the ikaite columns

**Fig 1:**
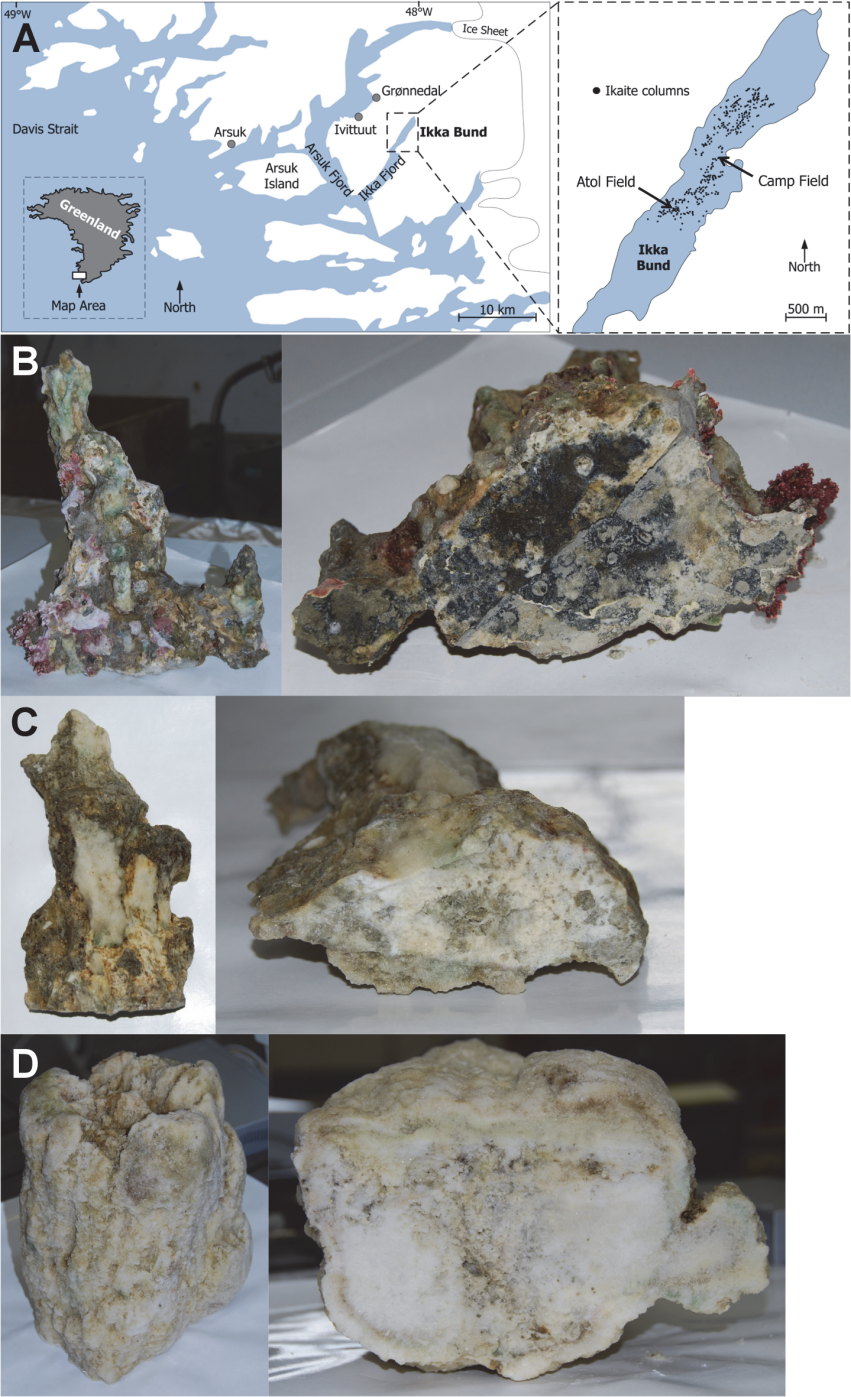
Map of the Ikka Fjord and images of ikaite columns used in this study. A, map of Southern Greenland showing the location of Ikka Fjord and the two areas, Atol and Camp Field, where material was collected for this study. B, C, D, surface (left) and cross-section (right) view of ikaite columns #1, #9 and #5, respectively (see S1 Table for details). All three harvested columns are approximately 40–60 cm in height.

Sections of individual ikaite columns were collected during the summer from two locations in the Ikka Fjord (Fig 1). Columns were selected based on differences in size and apparent age as estimated by both the colour and hardness of the ikaite material. Older columns are affected by partial recrystallisation of ikaite into monohydrocalcite and calcite, which forms a hardened cement-like material, whereas ikaite is soft and porous [22]. Although older columns are hardened structures, which could potentially trap and isolate dead or dormant microorganisms, most were still visibly porous to some extent and precipitation of new ikaite was observed at the surface of all collected columns, suggesting active transport of spring water (data not shown). The cross-sections of columns had a heterogeneous appearance, ranging from white to light grey with patches of dark grey, brown, black and green in both new and old columns (Fig 1). The surface of the older columns was similarly heterogeneous and showed extensive growth of coralline red algae as well as patches of newly precipitated ikaite ranging from white to green. Newer column surfaces were light grey or brown to green, suggesting that newly formed ikaite can be colonised by phototrophic organisms. For practical reasons, and due to the protected nature of the Ikka Fjord, only the tips of columns were collected. Since the ikaite columns are continuously growing structures, the samples used in this study are therefore likely to represent ikaite that is younger than the average ikaite column.

Some columns gave of a distinctive odour of hydrogen sulphide (H_2_S) when above water immediately after collection. This was also noted on a previous expedition [26], and is usually associated with the activity of sulphate-reducing bacteria (SRB) [4]. The sulphide concentration in ikaite columns has not been measured, but the observation that the column interior is often blackened, could be an indication of precipitated metal sulphides. Similarly, the black and anoxic sediments observed in some soda lakes have been taken as an indicator of the presence of sulphide and anaerobic populations of SRB [4, 6, 44].

Samples for DNA extraction were taken from column surfaces and cross-sections and classified as either ‘old’ or ‘new’ material based on the observations detailed above. Furthermore, samples were defined as being representative of the surface community when taken either directly from the surface or from cross-sections adjacent to the surface. Approximately 50% of all samples taken from older, hardened columns failed to yield any DNA as judged by spectrophotometric measurements and PCR amplification of 16S rRNA genes, suggesting a very low level of biomass in these samples (data not shown). A total of 30 interior and 18 surface samples, as well as three seawater and two sediment samples, were obtained for pyrosequencing. Details of individual columns and samples used for pyrosequencing are given in S1 Table.

### Pyrosequencing of 16S rRNA genes

Partial 16S rRNA gene sequences were amplified using primers flanking the V3 and V4 hypervariable regions of both bacteria and archaea. Two technical triplicates where prepared as sequencing controls by separate amplification and pyrosequencing of samples I11 and I39. Pyrosequencing of the 57 samples generated 654,635 high-quality sequences after trimming and quality filtering. Filtering of chimeric sequences and clustering at 97% identity resulted in 452,477 sequences in 18,410 operational taxonomic units (OTUs). Of these, 9,407 OTUs were represented by only a single sequence (singletons) and were discarded before further analysis leaving 9,003 OTUs in the dataset covering ikaite, seawater and sediment samples (S2 Table). Phylogeny was assigned to each OTU by comparison to the Greengenes database (http://greengenes.lbl.gov/). An overview of the combined frequency of all phylogenetic groups and of each identified OTU in both individual samples and in combinations of related samples can be found in S3 and S4 Tables, respectively.

### Microbial diversity and richness in the ikaite columns

The generated sequences and OTUs were used to estimate the richness and diversity of the ikaite community. It is worth noting that sequence data alone does not take into account the proportion of dead or dormant microorganisms and may thus overestimate the number of active bacteria participating in community functions. Bacteria have previously been cultivated directly from both new and old ikaite [26, 29], but the cultured diversity only covers a small part of the total diversity and the proportion of inactive microorganisms in old ikaite is unknown. Regardless, the relatively young material used in this study (see above) would suggest that the detected microorganisms have had a recent function in the column community.

**Fig 2:**
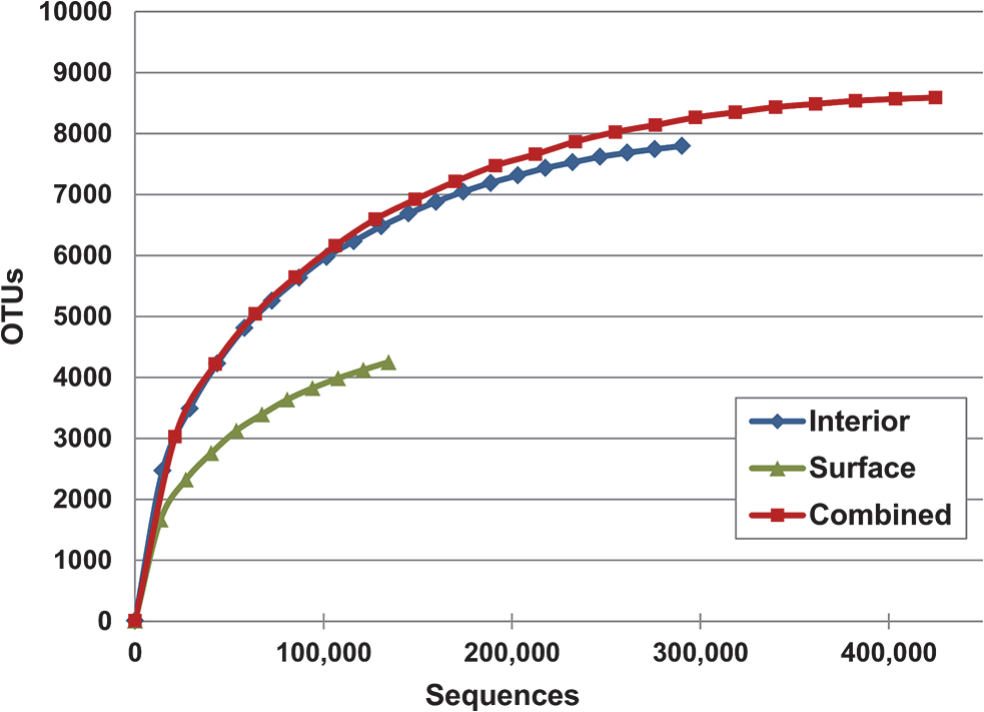
Rarefaction analysis of sequences from ikaite columns. OTUs were generated from the complete dataset by sequence clustering at 97% identity. Rarefaction curves were generated from OTUs present in samples of ikaite column interior or surface, as well as in both sample types (combined). Each data point is the average of 10 calculations based on separate subsamplings to the indicated number of sequences.

Rarefaction analysis of species (OTU) richness in a combined dataset of all samples taken from the ikaite columns resulted in a curve close to saturation at the 8,590 OTUs identified, suggesting that further sequencing effort is unlikely to significantly increase the number of observed OTUs in these samples. Rarefaction curves of interior and surface samples were approaching saturation at the 7,798 and 4,252 OTUs identified, respectively, indicating a significant OTU overlap between these two datasets (Fig 2). A total of 1,189 and 1,023 OTUs were identified from the seawater and sediment samples, respectively (Table 1).

**Table 1:** Shannon diversity index and the number of observed OTUs for combinations of sample type and ikaite age.

*Sample*	*Seqs*.	*Shannon*	*OTUs*
*Interior*	10,354	8.967	2,050
	All		7,798
*Surface*	10,354	7.690	1,449
	All		4,252
*Seawater*	10,354	6.508	962
	All		1,189
*Sediment*	10,354	7.764	1,023
	All		1,023
*Ikaite*, *all samples*	134,578	9.243	6,717
	All		8,590
*Interior*	134,578	9.300	6,515
*Surface*	134,578	7.907	4,252
*Interior*, *old ikaite*	62,361	9.070	4,727
*Interior*, *new ikaite*	62,361	8.683	3,906
*Surface*, *old ikaite*	62,361	8.200	3,508
*Surface*, *new ikaite*	62,361	6.712	1,974

All combinations of samples were subsampled to an even depth (sequences) before analysis. Values are the average of 10 separate subsamplings.

The total microbial diversity was estimated using the Shannon diversity index for various combinations of samples (Table 1). As suggested by the rarefaction analysis, the diversity was higher in the interior samples than in the surface samples, with Shannon indices of 9.300 and 7.907, respectively. A similar increase in diversity was observed in the alkaline Mono Lake, California, and Soap Lake, Washington, when going from the light-exposed, oxic surface waters to the anoxic bottom [45, 46]. In addition, both diversity and OTU richness was higher in old ikaite, both internally and at the surface, but it is possible that this is a consequence of an increased proportion of dead or dormant microorganisms in older ikaite, rather than an indicator of a more complex community. The microbial community in the ikaite columns was more diverse than both the seawater and sediment communities, challenging the notion that apparently hostile environments should be less diverse. A recent analysis of five Ethiopian soda lakes found that OTU richness was highest in the most alkaline and saline lake [7] and OTU richness in the ikaite columns appeared to be even higher. Although differences in sampling and technical and analytic approaches make a direct comparison difficult, the results suggest that the ikaite columns are home to a remarkably diverse microbial community.

### Surface versus interior community

**Fig 3:**
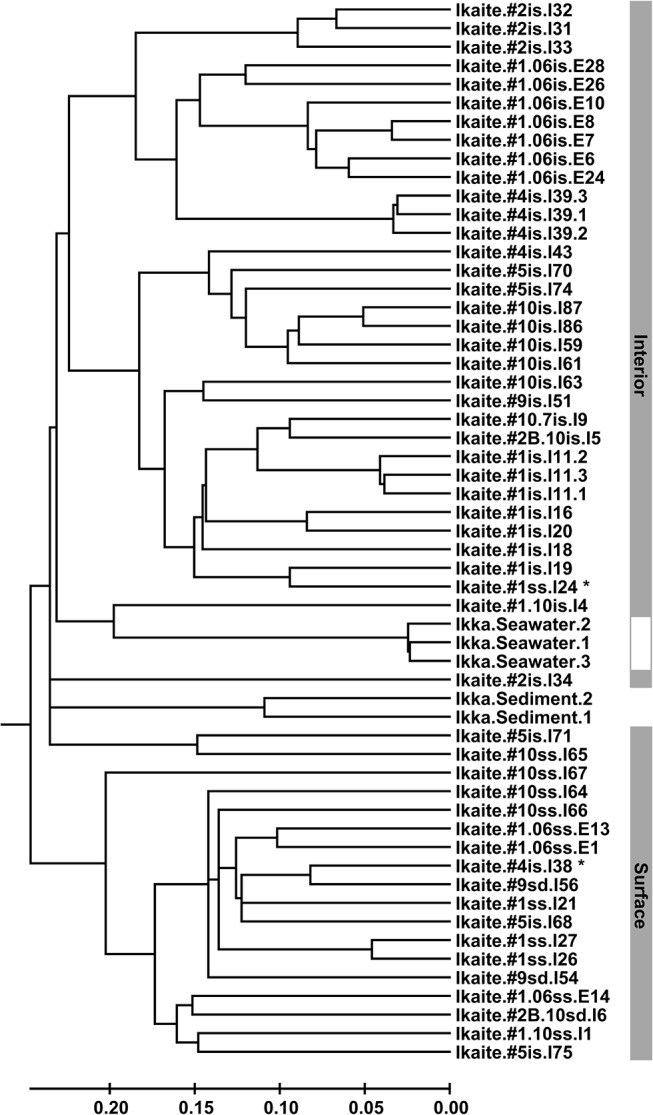
Dendrogram of all samples. Analysis of the overall community structure (beta-diversity) was carried out using average linkage cluster analysis (UPGMA) with the weighted UniFrac distance metric. The separate clustering of samples taken from ikaite column cross-sections (interior) and surfaces are indicated by the grey bars.

Differences in overall community composition (beta-diversity) were investigated using average linkage cluster analysis (UPGMA) and the weighted UniFrac distance metric [47]. The resulting dendrogram showed a clear separation between interior and surface samples, although some overlap was observed (Fig 3). Specifically, one interior sample from column #4 (sample I38) clustering with the surface samples was taken near the tip of a new column, which is likely to be actively growing, and the sample might thus better represent the surface community. Phototrophic organisms, which would naturally be present at the light-exposed surface, have previously been observed to be living inside the ikaite columns, concentrated near the surface [24]. A gradient of light, pH, salt, oxygen and nutrients between ikaite column water and the surrounding seawater might exist in the outer layers of an ikaite column making it difficult to precisely define a boundary between the surface and interior community. A surface sample from column #1 clustered with interior samples from the same column (sample I24). This sample was taken from an area encrusted by coralline red algae, which is likely to inhibit both the penetration of light and the mixing of waters, leading to near-surface conditions similar to those in the column interior. The tight clustering of the two technical triplicates (samples I11 and I39) indicates that the observed variation among the other samples is primarily biological (Fig 3).

Despite the clear differences in overall community structure, rarefaction analysis (see above) suggested a significant overlap in the presence of OTUs between surface and interior samples. This was confirmed by an analysis of the OTU distribution between these two sample types, which identified 3,460 shared OTUs representing 81.4% and 44.4% of all OTUs from the surface and interior datasets, respectively. While this does not take in to account the abundances of individual OTUs, it is in agreement with the observation that ikaite columns can grow up to 50 cm per year [16], which would lead to rapid envelopment and internalisation of the surface community. The constant flow of spring water through the columns could also be responsible for carrying bacteria from the interior to the surface. Despite the intervening seawater, this is also the most likely route by which new columns are colonised by the alkaline-adapted community. The prominent community overlap could also be a result of the sampling method and classification in relation to the geological development of the columns as discussed above. Some care must thus be taken when interpreting community data, especially when focusing on the cold- and alkaline-adapted community, which is expected to inhabit the interior of the columns.

### Overall taxonomic distribution

**Fig 4:**
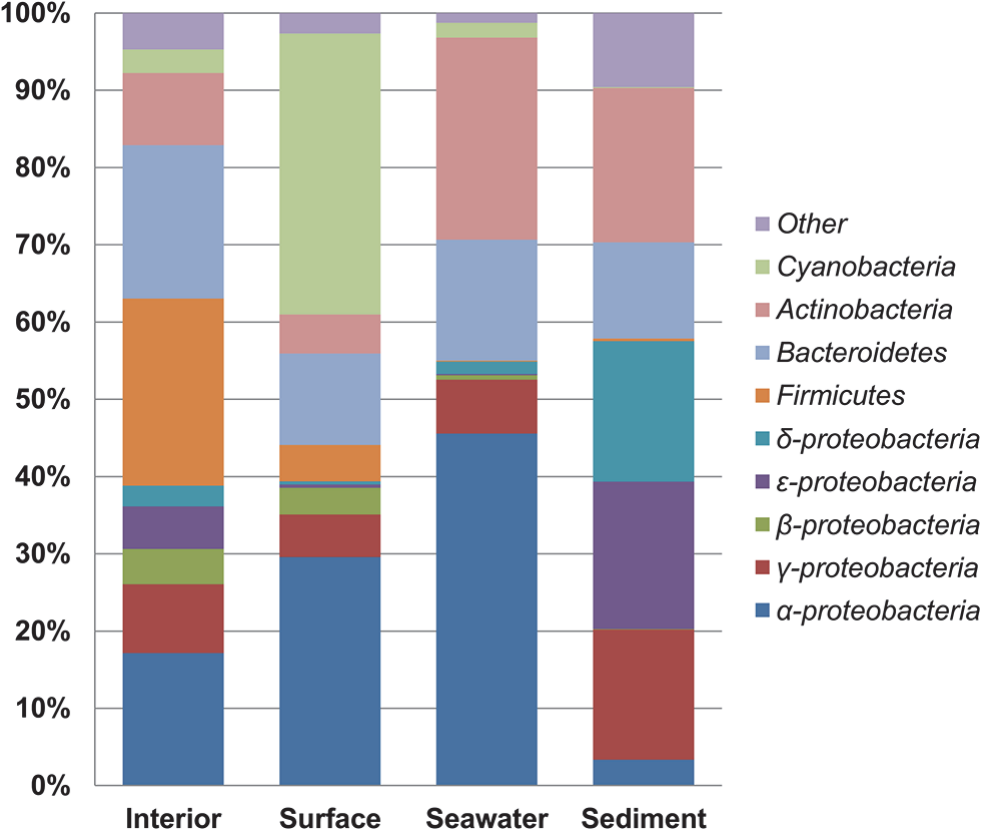
Distribution of phyla and proteobacterial classes in each sample type. The data represents the average abundance in groups of samples taken from cross-sections (interior) or surfaces of ikaite columns or from seawater or sediment in the Ikka Fjord.

In order to obtain a more accurate representation of the interior/surface community, two samples were reclassified based on the cluster analysis (Fig 3, see above). One interior sample was grouped with the surface samples (sample I38) and one surface sample was reclassified as an interior sample (sample I24). An analysis of the distribution of the most abundant bacterial phyla revealed that the ikaite columns are dominated by *Proteobacteria*, *Firmicutes*, *Bacteroidetes*, *Actinobacteria*, and *Cyanobacteria*, whereas the microbial diversity in seawater and sediment samples were mainly restricted to *Proteobacteria*, *Bacteroidetes*, and *Actinobacteria* (Fig 4). The most prominent differences between column interior and surface were observed for the *Firmicutes*, at 24.2% and 4.7%, and the *Cyanobacteria*, at 3.1% and 36.4%, respectively. Apart from *Cyanobacteria*, the surface was also abundant in *Alphaproteobacteria*, order *Rhodobacterales* (27.5%), which include numerous phototrophic genera (Table 2). The *Firmicutes* were almost exclusively of the class *Clostridia* (23.7%), orders *Natranaerobiales* (12.8%), *Clostridiales* (7.7%), and *Thermoanaerobacterales* (2.9%), most of which grow anaerobically [48]. Similarly, the *Bacteroidetes* identified in the column interior, primarily represented by the class *Bacteroidia*, order *Bacteroidales* (16.5%), are mostly anaerobic. The abundance of these orders suggests that the column interior is a largely anoxic environment, much like the lower layers and sediment of soda lakes, which are also abundant in anaerobic *Firmicutes* and *Bacteroidetes* [4, 45, 49, 50]. The anaerobic haloalkaliphilic members of the order *Natranaerobiales* were first identified in soda lakes of the Wadi An Natrun in Egypt and the abundant *Natranaerobiales* in the ikaite columns may represent low-saline, cold-adapted relatives of this newly described order [48]. *Proteobacteria* were abundant in all samples, although with significant differences in the distribution between classes (Table 2). *Alpha*-, *Beta*- and *Gammaproteobacteria* were present in both column interior and at the surface, while the *Delta*- and *Epsilonproteobacteria* where primarily found in the column interior and additionally, in very high numbers in sediment samples.

**Table 2:** The percentage frequency of the most abundant identifiable phyla, classes and orders.

*Phylum*	*Class*	*Order*	*Interior*	*Surface*	*Seawater*	*Sediment*
***Proteobacteria***			38.8	39.4	54.9	57.5
	*Alphaproteobacteria*		17.1	29.6	45.6	3.3
		*Rhodobacterales*	14.1	27.5	23.4	1.1
		*Rhizobiales*	1.7	1.5	0.3	2.2
		*Rickettsiales*	0.7	0.1	20.8	0.0
	*Gammaproteobacteria*		8.9	5.5	7.0	16.9
		*Chromatiales*	3.7	1.9	0.8	5.0
		*Thiotrichales*	1.5	0.5	0.7	5.0
		*Alteromonadales*	1.1	1.0	2.1	3.4
		*Oceanospirillales*	0.8	0.5	2.7	0.0
		*Marinicellales*	0.1	0.3	0.2	2.1
	*Betaproteobacteria*		4.5	3.5	0.6	0.1
		*Rhodocyclales*	4.1	3.2	0.0	-
	*Epsilonproteobacteria*		5.5	0.4	0.2	19.1
		*Campylobacterales*	5.5	0.4	0.1	19.0
	*Deltaproteobacteria*		2.7	0.4	1.6	18.2
		*Desulfobacterales*	1.6	0.1	1.0	11.8
		*Myxococcales*	0.2	0.1	0.4	4.5
***Firmicutes***			24.2	4.7	0.1	0.3
	*Clostridia*		23.7	4.6	0.1	0.3
		*Natranaerobiales*	12.8	2.8	0.0	0.0
		*Clostridiales*	7.7	1.7	0.0	0.3
		*Thermoanaerobacterales*	2.9	0.1	-	-
***Bacteroidetes***			19.8	11.8	15.6	12.5
	*Bacteroidia*		16.5	2.1	0.2	4.3
		*Bacteroidales*	16.5	2.1	0.2	4.3
	*Flavobacteriia*		2.0	4.6	15.0	7.9
		*Flavobacteriales*	2.0	4.6	15.0	7.9
	*Rhodothermi*		0.8	2.3	0.0	0.0
		*Rhodothermales*	0.8	2.3	0.0	0.0
	*Cytophagia*		0.4	2.6	0.1	0.1
		*Cytophagales*	0.4	2.6	0.1	0.1
***Actinobacteria***			9.3	5.0	26.2	20.0
	*Acidimicrobiia*		4.8	3.0	5.6	16.8
		*Acidimicrobiales*	4.8	3.0	5.6	16.8
	*Actinobacteria*		2.5	1.1	20.4	2.5
		*Actinomycetales*	1.9	1.1	20.4	2.4
	*Thermoleophilia*		1.3	0.7	0.1	0.6
		*Solirubrobacterales*	1.2	0.7	0.1	0.6
***Cyanobacteria***			3.1	36.4	1.9	0.1
	*Oscillatoriophycideae*		0.5	15.9	-	-
		*Oscillatoriales*	0.3	6.7	-	-
		*Chroococcales*	0.2	9.1	-	-
	*Synechococcophycideae*		1.2	10.2	0.0	-
		*Synechococcales*	1.2	9.0	0.0	-
	*Nostocophycideae*		0.0	2.6	-	-
		*Stigonematales*	0.0	2.5	-	-
	*Chloroplast*		1.3	7.5	1.9	0.1
***Chloroflexi***			1.3	0.1	0.2	1.2
***Tenericutes***			0.7	0.0	0.0	-
***Thermi***			0.6	0.3	-	-
***Verrucomicrobia***			0.2	0.6	0.5	0.5
***GN02***			0.2	0.3	0.0	0.1
***Gemmatimonadetes***			0.1	0.1	0.1	0.9
***OD1***			0.1	0.9	-	-
***Acidobacteria***			0.1	0.0	0.2	3.0
***WS3***			0.0	0.0	0.0	1.1
***Other***			1.1	0.2	0.1	1.3
	*Other*		6.0	3.4	1.5	8.3
		*Other*	12.1	14.6	5.6	12.0

Relatively few archaea were present in the ikaite columns and these were almost exclusively found in the column interior (0.17%; S3 Table). This is in contrast to observations of soda lake diversity where haloalkaliphilic archaea of the phylum *Euryarchaeota* are abundant and play potentially important roles in nutrient cycling [6, 50, 51]. The low temperature and salt concentration in the ikaite columns may exclude related alkaliphilic archaea and indeed, the majority of detected archaea were assigned to the genus *Nitrosopumilus* in the phylum *Crenarchaeota*, which is abundant in marine environments [52].

### Detailed phylogenetic analysis

The family *Rhodobacteraceae* of the *Alphaproteobacteria* was highly represented both internally and at the surface (Table 3), particularly in surface samples from new columns (35.9%; S3 Table). This family contains anoxygenic phototrophs, including the purple non-sulphur bacteria *Rhodobaca*, which was the most abundant genus identified in the ikaite columns (Table 3). The type strain *Rhodobaca bogoriensis* was isolated from the alkaline Lake Bogoria [53] and relatives of *Rhodobaca* have been identified in many soda lake environments (Table 4). Among other phototrophs was the Cyanobacterial family *Phormidiaceae* in the order *Oscillatoriales*, which was abundant at the column surface (6.7%; S3 Table). This family contains the genus *Arthrospira* (also known as *Spirulina*), an abundant phototroph and a key primary producer in African soda lakes [6]. Similar to soda lake ecosystems, these phototrophs could be responsible for primary production at the surface and provide carbon and nitrogen for subsurface populations of heterotrophic bacteria. *Rhodobaca*, *Arthrospira* and other free-living *Cyanobacteria* were almost absent from seawater samples (S3 Table), suggesting that these phototrophs specifically colonise the ikaite column surface. The potentially rapid colonisation by phototrophic organisms was evident in samples of newly formed ikaite, where *Rhodobaca* and the *Cyanobacteria* (including chloroplast sequences) accounted for 33.5% and 36.6% of all sequences, respectively (S3 Table).

**Table 3:** The percentage frequency of the most abundant identifiable families and genera.

*Order*	*Family*	*Genus*	*Interior*	*Surface*	*Seawater*	*Sediment*
***Alphaproteobacteria***						
*Rhodobacterales*	*Rhodobacteraceae*		13.74	26.66	23.35	1.08
		*Rhodobaca*	6.84	17.83	0.02	-
		*Loktanella*	1.77	2.78	1.99	0.01
		*Paracoccus*	1.53	1.09	0.02	-
		*Phaeobacter*	0.83	1.02	11.89	0.50
		*Octadecabacter*	0.51	0.77	4.14	0.07
*Rhizobiales*	*Phyllobacteriaceae*		0.66	0.92	0.07	0.38
	*Hyphomicrobiaceae*		0.55	0.13	0.24	1.75
*Rickettsiales*	*Pelagibacteraceae*		0.62	0.00	20.68	0.01
***Gammaproteobacteria***						
*Chromatiales*	*Ectothiorhodospiraceae*		3.52	1.86	0.04	-
		*Thioalkalivibrio*	3.24	1.75	0.04	-
*Thiotrichales*	*Piscirickettsiaceae*		1.12	0.47	0.67	4.80
		*Thioalkalimicrobium*	0.66	0.09	-	-
*Oceanospirillales*	*Oceanospirillaceae*		0.63	0.47	0.63	-
		*Marinomonas*	0.53	0.01	0.05	-
*Vibrionales*	*Vibrionaceae*		0.53	0.01	0.04	-
***Betaproteobacteria***						
*Rhodocyclales*	*Rhodocyclaceae*		4.14	3.24	0.04	-
		*Azoarcus*	3.85	3.19	0.04	-
***Epsilonproteobacteria***						
*Campylobacterales*	*Helicobacteraceae*		5.48	0.43	0.13	18.90
		*Sulfurimonas*	2.71	0.12	-	0.05
***Deltaproteobacteria***						
*Desulfobacterales*	*Desulfobacteraceae*		1.54	0.13	0.27	3.54
*Desulfuromonadales*	*Desulfuromonadaceae*		0.53	0.06	0.11	0.68
***Firmicutes***						
*Natranaerobiales*	*ML1228J-1*		7.50	0.30	0.02	-
	*Anaerobrancaceae*		2.55	2.47	0.01	0.01
		*Dethiobacter*	2.03	0.57	0.01	-
	*YAB3B13*		1.02	0.04	-	-
*Clostridiales*	*Clostridiaceae*		5.87	1.50	0.02	0.15
		*Tindallia*	3.73	0.39	0.01	-
		*Alkaliphilus*	1.39	0.68	-	-
		*Clostridium*	0.52	0.29	0.01	0.15
***Bacteroidetes***						
*Bacteroidales*	*ML635J-40*		13.35	1.78	0.07	-
*Flavobacteriales*	*Flavobacteriaceae*		1.63	3.81	13.18	7.52
*Rhodothermales*	*Balneolaceae*		0.82	2.32	-	-
***Actinobacteria***						
*Acidimicrobiales*	*C111*		2.52	1.94	4.18	6.79
*WCHB1-81*	*At425_EubF1*		0.52	0.00	-	0.10
***Cyanobacteria***						
*Synechococcales*	*Synechococcaceae*		1.17	8.98	0.01	-
		*Paulinella*	1.16	8.91	0.01	-
***Teniricutes***						
*Acholeplasmatales*	*Acholeplasmataceae*		0.55	0.02	0.01	-
***Thermi***						
*Deinococcales*	*Trueperaceae*		0.57	0.25	-	-
		*B-42*	0.54	0.25	-	-

Only groups with a frequency ≥ 0.5% in the ikaite column interior are included.

**Table 4:** Abundant phylogenetic groups identified in the ikaite column interior and examples of soda lakes with reported relatives.

*Genus*	*OTUs*	*Interior (%)*	*Surface (%)*	*Reported isolation source*	*Study*
*Rhodobaca*	340	6.84	17.8	Lake Bogoria, Kenya	[53]
				Wadi An Natrun, Egypt	[50]
				Soda lakes, Ethiopia	[7]
				Lonar Lake, India	[54]
				Lake Elmenteita, Kenya	[68]
				Soda lakes, Kenya	[69]
				Soap Lake, Washington, USA	[70]
				Mono Lake, California, USA	[45]
*Dethiobacter*	134	2.03	0.57	Soda lake, north-east Mongolia	[63]
				Lake Elmenteita, Kenya	[68]
*Desulfonatronum*	10	0.24	0.01	Mono Lake, California, USA	[71]
				Wadi An Natrun, Egypt	[50]
				Kulunda Steppe soda lakes, Altai, Russia	[61]
				Lake Khadin, Tuva, Russia	[72]
*Thioalkalimicrobium*	23	0.66	0.09	Soda lakes, Kenya and south-east Siberia	[60]
				Soda lake, north-east Mongolia	[73]
				Mono Lake, California, USA	[74]
				Soap Lake, Washington, USA	[75]
*Thioalkalivibrio*	80	3.24	1.75	Soda lakes, Kenya and south-east Siberia	[60]
				Mono Lake, California, USA	[74]
				Soda lakes, Ethiopia	[7]
				Wadi An Natrun, Egypt	[50]
				Soda lake, north-east Mongolia	[73]
				Lake Elmenteita, Kenya	[68]
				Soap Lake, Washington, USA	[46]
*Azoarcus*	76	3.85	3.19	Lake Arenguadi, Ethiopia	[7]
				Lonar Lake, India	[54]
*ML1228J-1 (family)*	338	7.5	0.3	Mono Lake, California, USA	[45]
				Wadi An Natrun, Egypt*	[50]
				Lonar Lake, India*	[54]
*ML635J-40 (family)*	431	13.4	1.78	Mono Lake, California, USA	[45]
				Lake Chitu and Arenguida, Ethiopia	[7]
				Lonar Lake, India*	[54]
*Tindallia*	124	3.73	0.39	Lake Magadi, Kenya	[76]
				Lake Texcoco, Mexico	[77]
				Mono Lake, California, USA	[78]
				Soda lakes, Kenya	[69]
				Soap Lake, Washington, USA	[46]
*Alkaliphilus*	77	1.39	0.68	Verkhnee Beloe soda lake, Siberia, Russia	[79]
				Lake Elmenteita, Kenya	[68]
				Lonar Lake, India	[54]
*Clostridium*	52	0.52	0.29	Verkhnee Beloe soda lake, Siberia, Russia	[80]
				Lake Elmenteita, Kenya	[68]
				Soda lakes, Kenya	[69]
				Soap Lake, Washington, USA	[46]
*Paracoccus*	77	1.53	1.09	Wadi An Natrun, Egypt	[50]
				Lonar Lake, India	[5]
				Soap Lake, Washington, USA	[46]
*Methylomicrobium*	15	0.26	0.1	Lake Shalla and Arenguadi, Ethiopia	[7]
				Lonar Lake, India	[81]
				Soda lakes, Kenya	[56]
				Soda lakes, south-east Siberia, Russia	[82]
*Marinomonas*	11	0.53	0.01	Lake Shalla, Ethiopia	[7]

The number of OTUs and the total abundance in samples from ikaite column interior and surface are given for each group.

*Identified by blast homology search using the most abundant OTUs.

Two major groups of bacteria in the column interior were assigned to the uncharacterised families *ML1228J-1* in the *Firmicutes* and *ML635J-40* in the *Bacteroidetes* (Table 3). Both families are based on sequences from uncultured bacteria from the lower anoxic layers of Mono Lake, California [45], but relatives have also been identified in other soda lakes (Table 4) and the most abundant ikaite OTUs assigned to these families were related (up to 97% identity, data not shown) to recently obtained sequences from a study of Lonar Lake sediments [54]. The function of these abundant groups in the ikaite columns is unknown, but the environment from which they were originally isolated and their phylogenetic affiliation would suggest that they are anaerobic. Similarly, the abundant family *Clostridiaceae* was mainly represented by the anaerobic alkaliphilic genera *Tindallia* and *Alkaliphilus* (Table 3). Relatives of these genera have been isolated from several soda lakes (Table 4). The ubiquitous genus *Clostridium*, which contains both alkaliphilic [4] and psychrophilic species [55], was also identified in the ikaite columns (Table 3).

Soda lake methanotrophs are dominated by alkaliphiles from the Gammaproteobacterial genus *Methylomicrobium*, which was also detected in the ikaite column interior (0.26%; S3 Table). They are obligate methylotrophs common in soda lake environments (Table 4) and may potentially participate in carbon cycling in the ikaite columns by degrading the products of the heterotrophic anaerobes, such as the *Clostridia* mentioned above [56].

OTUs classified to the genus *Azoarcus* (*Betaproteobacteria*) were abundant both internally and at the surface of columns (Table 3). *Azoarcus* has also been identified in low-saline soda lakes (Table 4) and the two closest relatives of the most abundant ikaite *Azoarcus* OTU were sequences isolated from low-saline alkaline lakes (>99% identity to GenBank acc. nos. KC358233 and HQ703857; data not shown), suggesting that alkali-tolerant members of this genera are less prominent in high-saline environments. The closest characterised relative was *Azoarcus taiwanensis* (97.6%), a denitrifying, sulphide-oxidising facultative alkaliphile [57].

The family *Helicobacteraceae* in the *Epsilonproteobacteria* was very abundant in the column interior and in particular in sediment samples, but whereas a significant number of sequences from the columns were assigned to the genus *Sulfurimonas*, this genus was virtually absent from sediment samples (Table 3), suggesting that the ikaite OTUs represent specific alkaline-adapted relatives of *Sulfurimonas*. Members of *Sulfurimonas* are chemolithoautotrophic sulphur-oxidisers abundant in marine sulphidic environments [58, 59] and while this genus has not previously been associated with alkaline habitats, other groups of sulphur-oxidisers are common in soda lakes. The families *Chromatiaceae* and *Ectothiorhodospiraceae* in the *Gammaproteobacteria* include the phototrophic purple sulphur bacteria, which use reduced sulphur compounds as electron donors. The majority of sequences assigned to these families were classified to the genus *Thioalkalivibrio*, which is not phototrophic, but includes alkaliphilic obligate chemolithoautotrophic sulphur-oxidising members [60]. *Thioalkalivibrio* was abundant in both interior and surface samples and appears to be universally present in soda lake environments along with another sulphur-oxidising genus, *Thioalkalimicrobium*, which was also found in the ikaite columns (Table 3; Table 4).

Recent studies have revealed an active microbial sulphur cycle in soda lakes and numerous bacteria participating in the cycle have been isolated and characterised [44]. Although the reported concentration of sulphate in ikaite column seep water (2.8–4.0 mM) [18] is low compared to many soda lakes, it is within the range measured for some soda lakes with active sulphate-reducing communities [61] and local variations in concentration may be possible due to biological activity. Sulphate-reducing *Deltaproteobacteria* appear to play an important role in the sulphur cycle in soda lakes [44, 62] and several orders of obligate anaerobic SRB (*Desulfobacterales*, *Desulfuromonadales* and *Desulfovibrionales*) were identified in the ikaite column interior and additionally, in very high numbers in sediment samples (Table 2; S3 Table). The OTU distribution was clearly distinct between the two sample types (S4 Table) and the majority of ikaite sequences were assigned to the families *Desulfobacteraceae* and *Desulfuromonadaceae* (Table 3). The alkaliphilic genus *Desulfonatronum* in the order *Desulfovibrionales* was almost exclusively found in the ikaite column interior (S3 Table) and members of this genus have previously been isolated from soda lakes (Table 4). In addition to the *Deltaproteobacteria*, the genus *Dethiobacter*, class *Clostridia*, was abundant in the ikaite column interior (2.0%; Table 3). The type strain, *Dethiobacter alkaliphilus*, is an obligate anaerobic chemolithoautotrophic alkaliphilic SRB originally isolated from soda lakes in north-east Mongolia [63]. Similar to many other SRB it is capable of reducing a range of sulphur compounds, such as thiosulphate, elemental sulphur and polysulfide, and can also grow by disproportionation of elemental sulphur [64]. In addition to the presence of both sulphur-oxidising and sulphate-reducing bacteria, the distinct smell of sulphide and the blackened interior observed for some columns (see above) suggests that sulphur is actively cycled in the ikaite columns.

### The ikaite columns as a cold alkaline environment

The taxonomic distribution of bacteria in the ikaite columns was found to be similar to that of studied soda lake environments, both in the abundance of specific phylogenetic groups (Table 4) and in the apparent division between a light-exposed phototrophic community and a largely anaerobic subsurface community. On the other hand, the ikaite column community was distinct from those found at the highly alkaline terrestrial and subterrestrial serpentinising sites, particularly in the absence of the abundant betaproteobacterial genus *Hydrogenophaga* [9–11]. Despite the location in a permanently cold marine environment, the ikaite community clearly differed from those occupying the surrounding seawater and sediment of the Ikka Fjord, as well as from recently studied Arctic marine environments [65–67].

The separation between column surface and interior is similar to the stratification or water/sediment interface seen for soda lakes with an upper light-exposed, oxygenated layer and a lower anoxic layer rich in sulphur compounds. Furthermore, the geochemical environment encountered by the microorganisms inhabiting the ikaite columns is, in some respects, similar to that of soda lakes where the concentration of CO_3_^2-^ exceeds that of Ca^2+^ leading to precipitation of calcite (CaCO_3_) and subsequent evaporative concentration of CO_3_^2-^ [6]. In the ikaite columns, Ca^2+^ from seawater and CO_3_^2-^ from spring water precipitate as ikaite and the high pH is maintained by continuous influx of alkaline spring water. In both cases, a high concentration of carbonate ions leads to an elevated pH. The similarities in geochemistry and community structure suggest that community function and nutrient cycling in these two environments could be overlapping. A notable exception is the absence of the organotrophic haloalkaliphilic archaea in the ikaite columns, which can develop to high densities in soda lakes [6].

The significant overlap with soda lake communities would suggest that the high pH and perhaps general geochemical environment is the main selective driver, meaning that the ikaite columns are primarily inhabited by cold-adapted alkaliphiles rather than alkaline-adapted psychrophiles. This makes the columns an obvious target for comparative studies of cold-adaptation and how bacteria adapt to survive combinations of extreme environmental factors.

## Conclusion

In-depth pyrosequencing of 16S rRNA genes from the ikaite columns and the surrounding Ikka Fjord revealed a surprisingly diverse, distinct bacterial community inhabiting the ikaite columns. A clear difference between the phylogenetic groups identified at the surface and in the interior of the columns was observed. While the surface was dominated by primary producers, such as anoxygenic phototrophic bacteria and *Cyanobacteria*, the interior appeared to be a largely anoxic environment populated by anaerobic heterotrophs and chemolithotrophs participating in cycling of sulphur compounds. The functional separation between surface and subsurface is also present in classical soda lakes and many of the most abundant phylogenetic groups identified in the ikaite columns were related to common groups found in soda lake environments. The apparent analogy to a cold soda lake implies that isolation of new alkaliphilic low-temperature species could be a valuable tool for studying the evolution of cold-adaptation. In addition, the unique combination of low temperature and high pH makes the columns a prime target for bioprospecting studies aimed at identifying cold- and alkaline-active enzymes.

## Supporting Information

S1 Table Details of all ikaite columns and samples successfully used for pyrosequencing. Columns were classified as ‘old’ or ‘new’ based on the observations described in the main text. Samples were further classified as ‘interior sample (is)’ when taken from a cross-section, ‘surface sample (ss)’ when taken from the top 5 mm at the surface, and ‘surface drilling (sd)’ when taken by drilling 1–2 cm in from the surface.
(DOCX)

S2 Table Selected pyrosequencing statistics. Samples are named by column number (#), sample type (is, ss, sd) and sample number. See S1 Table for sample details.
(DOCX)

S3 Table Combined percentage frequency of genus-level taxonomic groups identified in the ikaite columns and Ikka Fjord. (XLSX)

S4 Table Percentage frequencies of all OTUs identified in the ikaite columns and Ikka Fjord. (XLSX)
