# Supplementary material for: Microbial Diversity in a Permanently Cold and Alkaline Environment in Greenland
Source: PLoS One. 2015 Apr 27;10(4):e0124863. doi: 10.1371/journal.pone.0124863 (PMC4411134; doi:10.1371/journal.pone.0124863)
Supplement: S1 Table — Columns were classified as ‘old’ or ‘new’ based on the observations described in the main text. Samples were further classified as ‘interior sample (is)’ when taken from a cross-section, ‘surface sample (ss)’ when taken from the top 5 mm at the surface, and ‘surface drilling (sd)’ when taken by drilling 1–2 cm in from the surface. (DOCX) [file pone.0124863.s001.docx]

| ***Column*** | ***Column details*** | ***Samples*** | ***Sample details*** |
| --- | --- | --- | --- |
| #1 | Old ikaite  Collected 2011 | Ikaite.#1is.I11.1  Ikaite.#1is.I11.2  Ikaite.#1is.I11.3  Ikaite.#1is.I16  Ikaite.#1is.I18  Ikaite.#1is.I19  Ikaite.#1is.I20  Ikaite.#1ss.I21  Ikaite.#1ss.I24  Ikaite.#1ss.I26  Ikaite.#1ss.I27 | Internal, pyrosequencing triplicate  Internal, pyrosequencing triplicate  Internal, pyrosequencing triplicate  Internal  Internal  Internal  Internal  Surface  Surface  Surface, newly formed ikaite, green  Surface, newly formed ikaite, green |
| #2 | New ikaite  Collected 2011 | Ikaite.#2is.I31  Ikaite.#2is.I32  Ikaite.#2is.I33  Ikaite.#2is.I34 | Internal  Internal  Internal  Internal |
| #4 | New ikaite  Collected 2011 | Ikaite.#4is.I38  Ikaite.#4is.I39.1  Ikaite.#4is.I39.2  Ikaite.#4is.I39.3  Ikaite.#4is.I43 | Internal  Internal, pyrosequencing triplicate  Internal, pyrosequencing triplicate  Internal, pyrosequencing triplicate  Internal |
| #5 | New ikaite formed after a cut in 2008  Collected 2011 | Ikaite.#5is.I68  Ikaite.#5is.I70  Ikaite.#5is.I71  Ikaite.#5is.I74  Ikaite.#5is.I75 | Surface, brown band 1 cm from the surface  Internal  Surface, brown/green band 1 cm from the surface  Internal  Surface, green band 1 cm from the surface |
| #9 | Old ikaite  Collected 2011 | Ikaite.#9is.I51  Ikaite.#9sd.I54  Ikaite.#9sd.I56 | Internal  Surface, drilled 2 cm  Surface, drilled 2 cm |
| #10 | Old ikaite  Collected 2011 | Ikaite.#10is.I59  Ikaite.#10is.I61  Ikaite.#10is.I63  Ikaite.#10ss.I64  Ikaite.#10ss.I65  Ikaite.#10ss.I66  Ikaite.#10ss.I67  Ikaite.#10is.I86  Ikaite.#10is.I87 | Internal  Internal  Internal  Surface, newly formed ikaite, white/green  Surface, newly formed ikaite, brown/green  Surface  Surface  Internal  Internal |
| #1.06 | Old ikaite, frozen  Collected 2006 | Ikaite.#1.06ss.E1  Ikaite.#1.06is.E6  Ikaite.#1.06is.E7  Ikaite.#1.06is.E8  Ikaite.#1.06is.E10  Ikaite.#1.06ss.E13  Ikaite.#1.06ss.E14  Ikaite.#1.06is.E24  Ikaite.#1.06is.E26  Ikaite.#1.06is.E28 | Surface  Internal  Internal  Internal  Internal  Surface  Surface  Internal  Internal  Internal |
| #1.10 | Old ikaite, frozen  Collected 2010 | Ikaite.#1.10ss.I1  Ikaite.#1.10is.I4 | Surface  Internal |
| #10.7 | Old ikaite, frozen  Collected 2007 | Ikaite.#10.7is.I9 | Internal |
| #2B.10 | Old ikaite, frozen  Collected 2010 | Ikaite.#2B.10is.I5  Ikaite.#2B.10sd.I6 | Internal  Surface, drilled 1.5 cm |
| Seawater | Collected 2011 | Ikka.Seawater.1  Ikka.Seawater.2  Ikka.Seawater.3 | -  -  - |
| Sediment | Collected 2011 | Ikka.Sediment.1  Ikka.Sediment.2 | Atol Field  Camp Field |

**S1 Table. Details of all ikaite columns and samples successfully used for pyrosequencing.** Columns were classified as ‘old’ or ‘new’ based on the observations described in the main text. Samples were further classified as ‘internal sample (is)’ when taken from a cross-section, ‘surface sample (ss)’ when taken from the top 5 mm at the surface, and ‘surface drilling (sd)’ when taken by drilling 1-2 cm in from the surface.
