# Supplementary material for: Microbial Diversity in a Permanently Cold and Alkaline Environment in Greenland
Source: PLoS One. 2015 Apr 27;10(4):e0124863. doi: 10.1371/journal.pone.0124863 (PMC4411134; doi:10.1371/journal.pone.0124863)
Supplement: S2 Table — Samples are named by column number (#), sample type (is, ss, sd) and sample number. See S1 Table for sample details. (DOCX) [file pone.0124863.s002.docx]

| ***After trimming and quality filtering*** | | |  |  |
| --- | --- | --- | --- | --- |
| *Sequences* | 654,635 |  |  |  |
| *Average length* | 383.8 |  |  |  |
|  |  |  |  |  |
| ***After chimera and singleton filtering*** | | |  |  |
| *OTU clustering (identity)* | *97%* |  |  |  |
| *Sequences* | 452,477 |  |  |  |
| *OTUs* | 9,003 |  |  |  |
| *Discarded singletons* | 9,407 |  |  |  |
|  |  |  |  |  |
| ***Sequences per sample*** | | |  | |
| *OTU clustering percentage* | *97%* |  |  |  |
| Ikaite.#1is.I11.1 | 12,519 |  | Ikaite.#10is.I61 | 8,190 |
| Ikaite.#1is.I11.2 | 7,119 |  | Ikaite.#10is.I63 | 10,973 |
| Ikaite.#1is.I11.3 | 10,683 |  | Ikaite.#10is.I86 | 6,823 |
| Ikaite.#1is.I16 | 7,470 |  | Ikaite.#10is.I87 | 7,613 |
| Ikaite.#1is.I18 | 7,245 |  | Ikaite.#10ss.I64 | 10,007 |
| Ikaite.#1is.I19 | 5,560 |  | Ikaite.#10ss.I65 | 10,798 |
| Ikaite.#1is.I20 | 9,231 |  | Ikaite.#10ss.I66 | 4,237 |
| Ikaite.#1ss.I21 | 6,440 |  | Ikaite.#10ss.I67 | 7,232 |
| Ikaite.#1ss.I24 | 6,266 |  | Ikaite.#1.06is.E10 | 7,427 |
| Ikaite.#1ss.I26 | 8,198 |  | Ikaite.#1.06is.E24 | 12,928 |
| Ikaite.#1ss.I27 | 7,722 |  | Ikaite.#1.06is.E26 | 10,250 |
| Ikaite.#2is.I31 | 8,016 |  | Ikaite.#1.06is.E28 | 12,708 |
| Ikaite.#2is.I32 | 10,671 |  | Ikaite.#1.06is.E6 | 8,403 |
| Ikaite.#2is.I33 | 7,419 |  | Ikaite.#1.06is.E7 | 8,216 |
| Ikaite.#2is.I34 | 8,840 |  | Ikaite.#1.06is.E8 | 8,175 |
| Ikaite.#4is.I38 | 6,695 |  | Ikaite.#1.06ss.E1 | 6,810 |
| Ikaite.#4is.I39.1 | 3,827 |  | Ikaite.#1.06ss.E13 | 5,754 |
| Ikaite.#4is.I39.2 | 6,948 |  | Ikaite.#1.06ss.E14 | 5,914 |
| Ikaite.#4is.I39.3 | 21,235 |  | Ikaite.#1.10is.I4 | 6,182 |
| Ikaite.#4is.I43 | 7,546 |  | Ikaite.#1.10ss.I1 | 5,704 |
| Ikaite.#5is.I68 | 8,489 |  | Ikaite.#10.7is.I9 | 3,219 |
| Ikaite.#5is.I70 | 9,205 |  | Ikaite.#2B.10is.I5 | 7,053 |
| Ikaite.#5is.I71 | 8,403 |  | Ikaite.#2B.10sd.I6 | 6,897 |
| Ikaite.#5is.I74 | 7,453 |  | Ikka.Seawater.1 | 6,199 |
| Ikaite.#5is.I75 | 8,744 |  | Ikka.Seawater.2 | 5,694 |
| Ikaite.#9is.I51 | 5,968 |  | Ikka.Seawater.3 | 5,396 |
| Ikaite.#9sd.I54 | 8,853 |  | Ikka.Sediment.1 | 5,650 |
| Ikaite.#9sd.I56 | 8,110 |  | Ikka.Sediment.2 | 4,704 |
| Ikaite.#10is.I59 | 8,446 |  |  |  |

**S2 Table. Selected pyrosequencing statistics.** Samples are named by column number (#), sample type (is, ss, sd) and sample number. See S1 Table for sample details.
